# Single‐Stage Non‐Exposed Endoscopic Wall‐Inversion Surgery and Laparoscopic Pylorus‐Preserving Gastrectomy for a Gastric Leiomyoma in the Cardia and Early Gastric Cancer in the Middle Stomach: A Case Report

**DOI:** 10.1111/ases.70211

**Published:** 2025-12-14

**Authors:** Takayuki Morita, Makoto Ansai, Rikiya Kamba, Keisuke Fukushima, Kazutomi Takahashi, Mineto Ohta, Kenji Namiki

**Affiliations:** ^1^ Department of General Surgery Tohoku University Hospital Sendai Japan; ^2^ Department of Surgery Osaki Citizen Hospital Osaki Japan

**Keywords:** gastrectomy, gastrointestinal endoscopy, laparoscopy

## Abstract

Non‐exposed endoscopic wall‐inversion surgery (NEWS) is a method of endoscopic full‐thickness resection without transluminal access. We report a case of simultaneous NEWS and laparoscopic pylorus‐preserving gastrectomy (LPPG) for a gastric leiomyoma in the cardia and early gastric cancer (EGC) in the middle stomach. A 56‐year‐old woman had a 15 mm intraluminal submucosal tumor (SMT) on the posterior wall of the cardia and a type 0–IIc lesion on the greater curvature of the middle stomach. Biopsy suggested the SMT was a leiomyoma or gastrointestinal stromal tumor, and the type 0–IIc lesion was poorly differentiated adenocarcinoma, consistent with EGC. The SMT was located approximately 10 mm distal to the esophagogastric junction, and total gastrectomy was initially considered. However, to avoid extensive gastric resection and preserve gastric function, we performed LPPG for the EGC followed by NEWS for the SMT, achieving minimally invasive, function‐preserving surgery. The patient was discharged uneventfully on postoperative day 8.

## 1 Introduction

Non‐exposed endoscopic wall‐inversion surgery (NEWS), a variant of laparoscopic and endoscopic cooperative surgery (LECS), allows non‐exposed full‐thickness gastric resection with the minimum necessary negative margins while reducing the risks of intraperitoneal contamination and tumor cell dissemination [1]. These advantages have broadened indications beyond submucosal tumors (SMTs), including those with mucosal involvement, to early gastric cancers (EGCs) in which endoscopic submucosal dissection (ESD) is technically difficult. Accordingly, it has been increasingly adopted as a function‐preserving local resection technique. Here, we report a case in which NEWS and laparoscopic pylorus‐preserving gastrectomy (LPPG) were performed simultaneously—NEWS for a gastric leiomyoma in the cardia and LPPG for EGC in the middle stomach. Given the SMT's proximity to the esophagogastric junction (EGJ), total gastrectomy would typically be considered; however, the combined approach enabled minimally invasive, function‐preserving surgery.

## 2 Case Presentation

**FIGURE 1:**
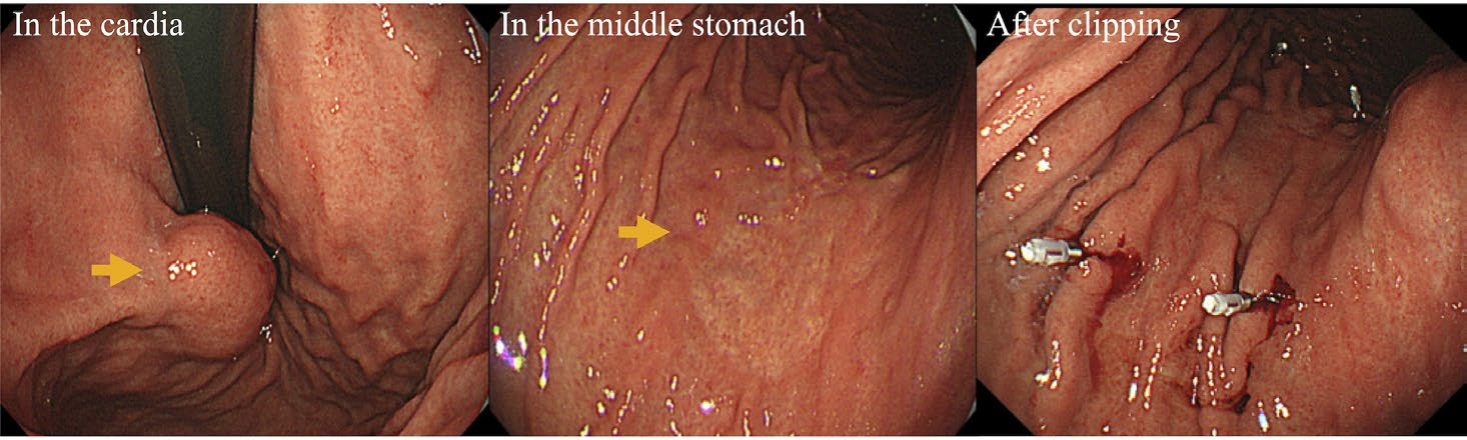
Upper gastrointestinal endoscopy. A submucosal tumor on the posterior wall of the cardia, showing intraluminal growth (arrow), and a type 0–IIc lesion on the greater curvature of the middle stomach (arrow); the oral and anal sides of the 0–IIc lesion are marked after clip placement.

**FIGURE 2:**
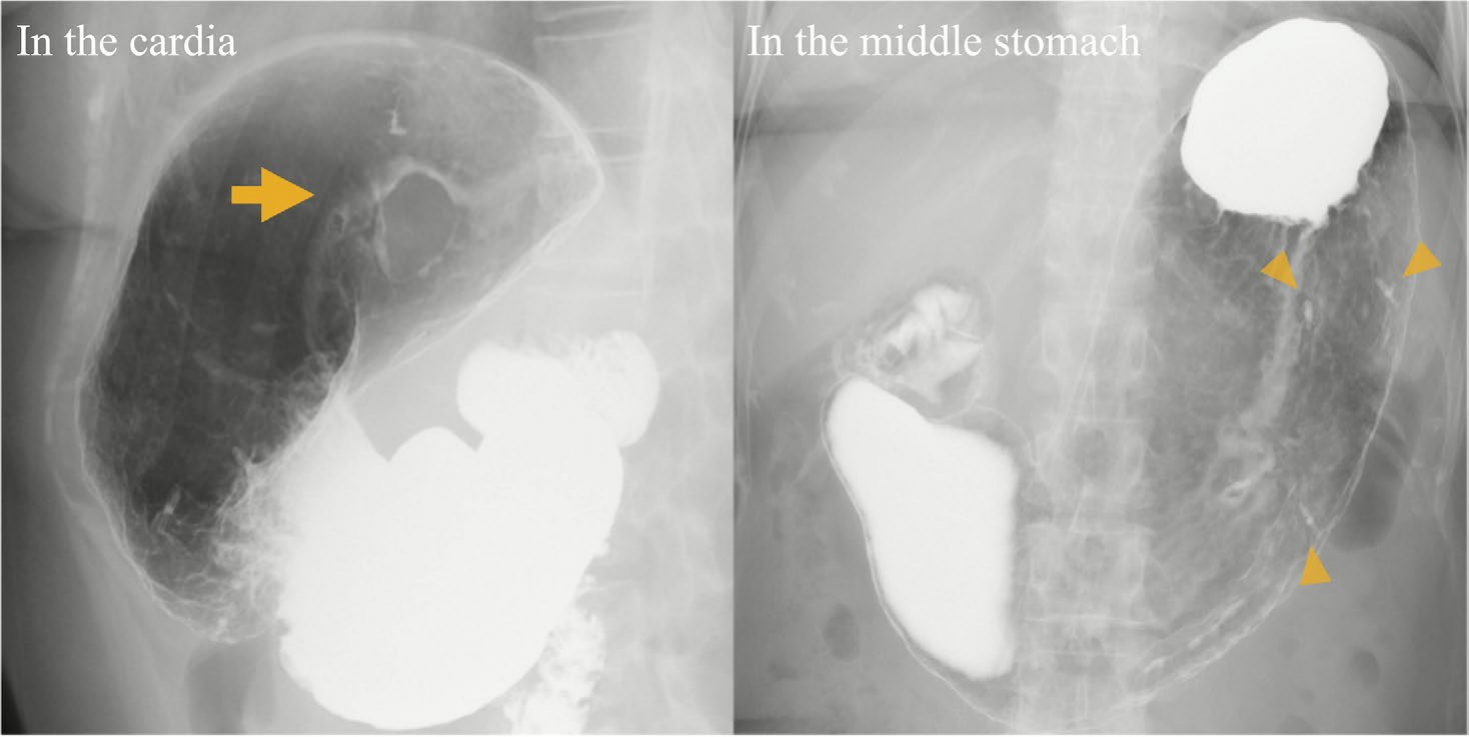
Upper gastrointestinal series. A smooth‐surfaced filling defect in the cardia is seen (arrows). Clips mark the oral and anal sides of the type 0–IIc lesion on the greater curvature of the middle stomach (arrowheads).

**FIGURE 3:**
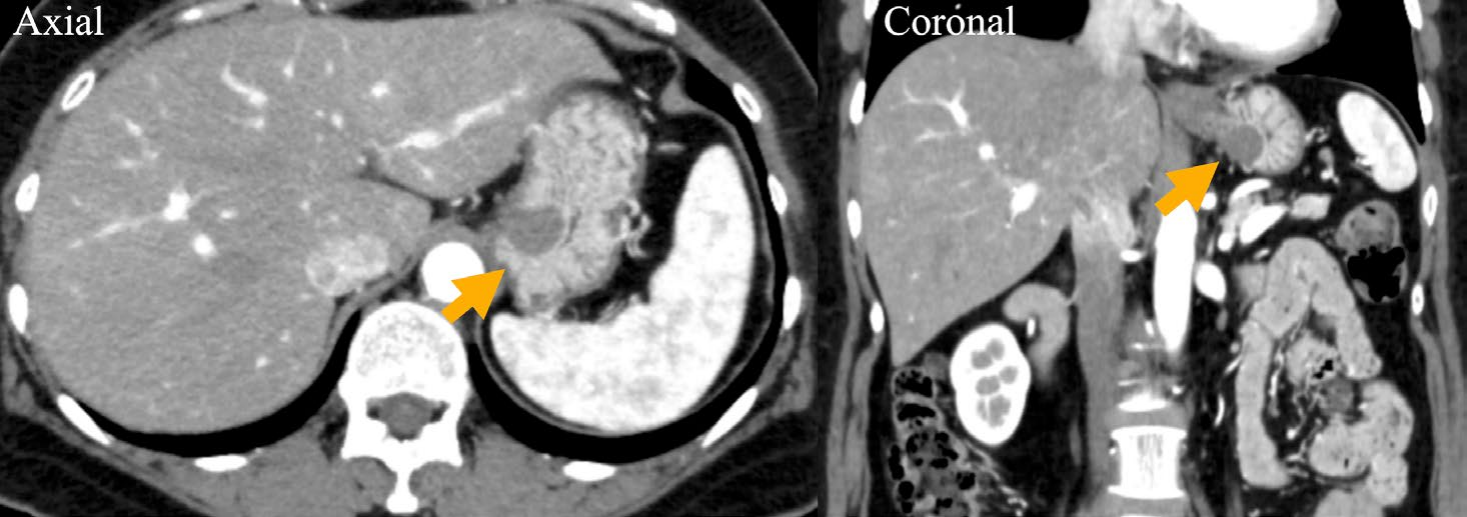
Contrast‐enhanced computed tomography. An intramural submucosal tumor in the cardia is seen (arrow). The type 0–IIc lesion on the greater curvature is not delineated. No regional lymph node enlargement or distant metastasis is evident.

**FIGURE 4:**
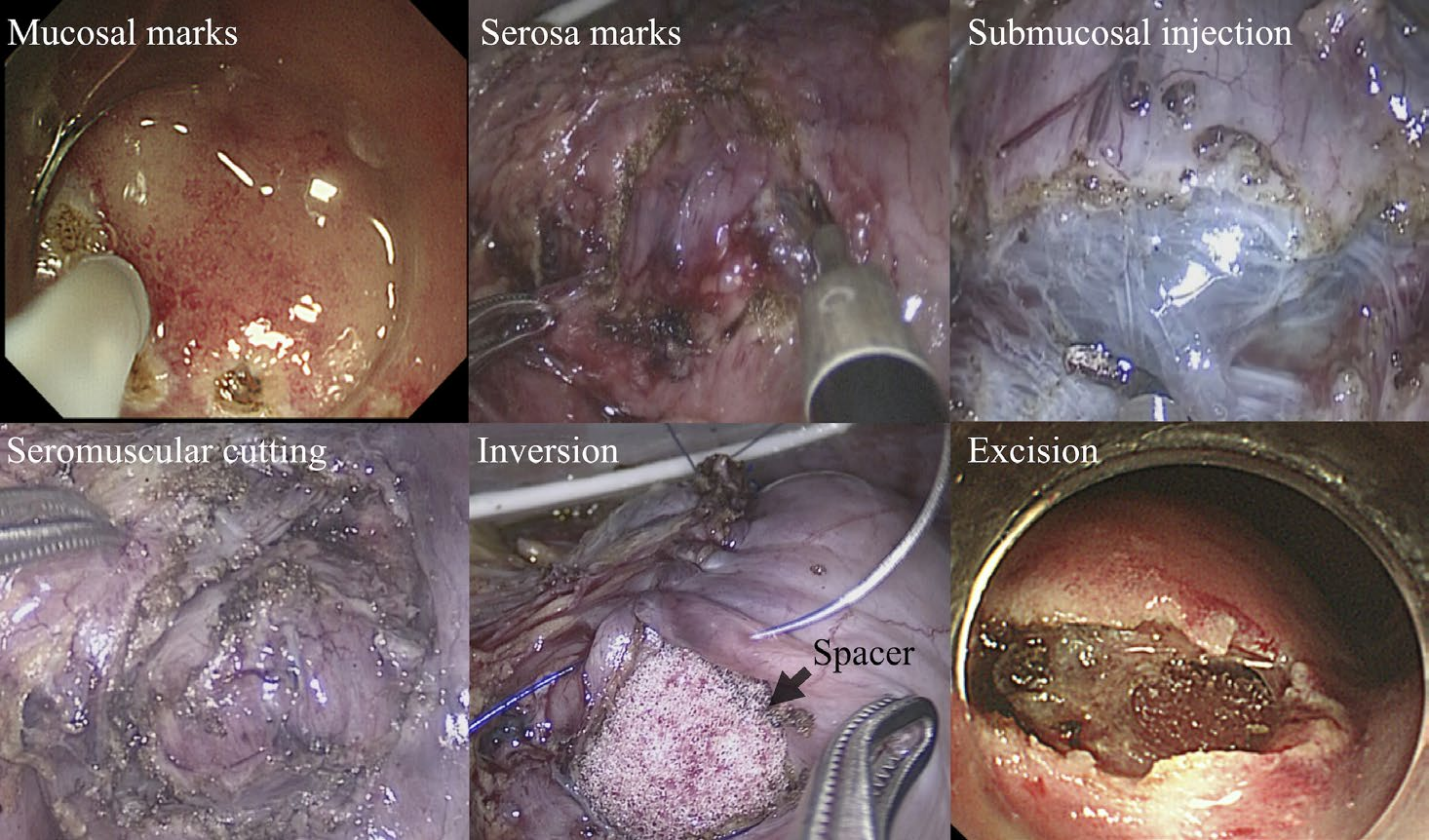
Procedure of non‐exposed endoscopic wall‐inversion surgery (NEWS). Endoscopic mucosal and corresponding laparoscopic serosal markings were created. After endoscopic submucosal injection of sodium hyaluronate solution (MucoUP), a circumferential seromuscular incision was made; the tumor‐bearing full‐thickness gastric wall was inverted into the lumen with spacer placement, and the seromuscular layer was closed to maintain non‐exposure. Finally, the SMT was excised endoscopically.

A 56‐year‐old woman was referred for evaluation of anemia. Upper gastrointestinal endoscopy identified a 15 mm intraluminal SMT on the posterior wall of the cardia, ~10 mm distal to the EGJ (Figure 1). Endoscopic ultrasonography showed a homogeneous, hypoechoic mass arising from the muscularis propria. Endoscopic ultrasound‐guided fine‐needle aspiration revealed spindle‐cell proliferation with minimal atypia; immunohistochemistry was strongly positive for smooth‐muscle markers (α‐SMA, desmin), favoring leiomyoma; however, weak c‐kit/DOG1 staining meant gastrointestinal stromal tumor (GIST) could not be excluded. Additionally, a 25 mm type 0–IIc lesion was identified on the greater curvature of the middle stomach (Figure 1). Biopsy revealed poorly differentiated adenocarcinoma (tub2 + por), suggesting EGC with submucosal invasion. Laboratory tests (complete blood count, liver/renal function) were unremarkable; tumor markers (CEA, CA 19–9) were within normal limits. Upper gastrointestinal series demonstrated a smooth‐surfaced filling defect in the cardia, and confirmed clips placed on the oral and anal sides of the 0–IIc lesion (Figure 2). Contrast‐enhanced computed tomography (CT) showed no lymph node enlargement or distant metastasis (Figure 3). Accordingly, the clinical diagnosis was leiomyoma or GIST together with EGC (cT1bN0M0; cStage IA), according to the Union for International Cancer Control (UICC) TNM classification (8th edition). To avoid extensive gastric resection and preserve gastric function, we planned a single‐stage procedure—LPPG for the cancer followed by NEWS for the SMT—in collaboration with the gastroenterology team. Surgery commenced with a five‐port laparoscopic approach. D1+ lymph node dissection was performed first. To secure an adequate endoscopic working space, the distal side of the stomach was transected, after which NEWS was undertaken (Figure 4). The SMT was marked endoscopically, and corresponding serosal marks were placed laparoscopically around the lesion. After endoscopic submucosal injection of sodium hyaluronate solution (MucoUP, Seikagaku Corporation, Tokyo, Japan), a circumferential seromuscular incision was created laparoscopically. The tumor‐bearing full‐thickness gastric wall was inverted into the lumen, a spacer was inserted, and the seromuscular layer was sutured closed over it; the SMT was then excised endoscopically. The SMT and spacer were pushed into the resected portion of the stomach; the proximal side of the stomach was subsequently resected, and the combined specimen—including the EGC lesion—was extracted through the umbilical incision. Reconstruction was completed by intracorporeal delta‐shaped gastrogastrostomy. The operative time was 5 h and 10 min, and blood loss was minimal. Pathological examination showed a poorly differentiated adenocarcinoma measuring 37 × 22 mm (0–IIc), classified as pT1b2 (SM2, 1 mm), pN1, Ly1a, V0, pPM0, pDM0, pStage IB (UICC TNM, 8th edition). The SMT was a 19 mm spindle‐cell lesion with low proliferative activity (Ki‐67 < 1%), and was resected with negative margins (R0). Immunohistochemistry was positive for α‐SMA, desmin, and h‐caldesmon, consistent with gastric leiomyoma. The postoperative course was uneventful; she was discharged on postoperative day 8 and remains recurrence‐free at 4.5 years.

## 3 Discussion

LECS was developed for gastric SMTs without mucosal involvement to minimize the extent of gastric resection; however, entering the gastric lumen raises concerns about intraperitoneal contamination and potential tumor cell dissemination. NEWS addresses these risks by inverting the lesion and closing the seromuscular layer before endoscopic excision, enabling full‐thickness resection with the minimum necessary negative margins [1]. Indications now include selected EGCs in which ESD is technically difficult; feasibility combined with sentinel node biopsy in nodal‐risk lesions has been reported, suggesting future expansion [2].

In this case, a cardia SMT and an EGC in the middle stomach coexisted. Because the SMT was ~10 mm distal to the EGJ, total gastrectomy was initially considered. After discussion with gastroenterology, we chose single‐stage LPPG followed by NEWS to preserve function and avoid extensive resection. As another function‐preserving option, proximal gastrectomy (PG) could have enabled en bloc resection; however, because the EGC was predominantly in the middle stomach, PG risked an inadequate distal margin or a small remnant stomach, increasing the likelihood of postoperative reflux and impaired reservoir function [3]. Among non‐exposure LECS variants, CLEAN‐NET and closed LECS were also considered [4, 5, 6]. CLEAN‐NET entails stapled full‐thickness resection, which—near the EGJ—risks cardia deformity/stenosis and over‐resection. Closed LECS resembles NEWS but starts with an endoscopic mucosal incision and ends with endoscopic full‐thickness transection, enabling precise margin setting. In our case, the SMT protruded intraluminally, so mucosal margins were easy to delineate; moreover, NEWS centers on laparoscopic steps before endoscopic excision and matched our LPPG‐first sequence.

Performing D1+ dissection and transecting the distal stomach first increased proximal mobility and created an adequate endoscopic working space. This mobility allowed rotation of the cardia for adequate exposure, enabling inversion and seromuscular closure without excessive tension. Although fundic (fornix) mobilization is commonly undertaken for posterior‐cardia NEWS [6], it was not required in our sequence. If exposure is still insufficient or the EGJ is torqued, selective fundic (fornix) mobilization (e.g., division of the short gastric vessels) should be considered. In addition, for efficiency, we also adopted single‐step retrieval; the combined specimen—the EGC lesion, the SMT, and the spacer—was extracted through the umbilical incision, obviating transoral retrieval.

In conclusion, LPPG and NEWS provided minimally invasive, function‐preserving management for synchronous lesions in distinct gastric regions. When early malignancies make gastric function difficult to preserve due to location or coexisting lesions, NEWS can serve as a valuable approach to achieve optimal outcomes.

## Ethics Statement

All procedures complied with the Declaration of Helsinki. Written informed consent for publication was obtained from the patient.

## Conflicts of Interest

The authors declare no conflicts of interest.

## Data Availability

The data that support the findings of this study are available on request from the corresponding author. The data are not publicly available due to privacy or ethical restrictions.
